# Association between Daily Physical Activity and Locomotive Syndrome in Community-Dwelling Japanese Older Adults: A Cross-Sectional Study

**DOI:** 10.3390/ijerph19138164

**Published:** 2022-07-03

**Authors:** Yoshihiko Ishihara, Hayao Ozaki, Takashi Nakagata, Toshinori Yoshihara, Toshiharu Natsume, Tomoharu Kitada, Masayoshi Ishibashi, Pengyu Deng, Yasuyuki Yamada, Hiroyuki Kobayashi, Shuichi Machida, Hisashi Naito

**Affiliations:** 1 School of Science and Technology for Future Life, Tokyo Denki University, Tokyo 120-8551, Japan; ishihara@mail.dendai.ac.jp; 2 Faculty of Health Sports Science, Juntendo University, Chiba 270-1695, Japan; ozaki-h@tokaigakuen-u.ac.jp (H.O.); takashi.nakagata@gmail.com (T.N.); t-yoshih@juntendo.ac.jp (T.Y.); deng@juntendo.ac.jp (P.D.); yayamada@juntendo.ac.jp (Y.Y.); hrkoba1@gmail.com (H.K.); hnaitou@juntendo.ac.jp (H.N.); 3 School of Sport and Health Science, Tokai Gakuen University, Miyoshi 470-0207, Japan; 4 Department of Physical Activity Research, National Institutes of Biomedical Innovation, Health and Nutrition (NIBIOHN), Tokyo 162-8636, Japan; 5 School of Medicine, Tokai University, Isehara 259-1193, Japan; nt554270@tsc.u-tokai.ac.jp; 6 COI Project Center, Juntendo University, Tokyo 113-8421, Japan; masa.tkr.ishibashi@gmail.com; 7 Faculty of Business Administration, Seijoh University, Miyoshi 476-8588, Japan; kitada@seijoh-u.ac.jp; 8 Graduate School of Health and Sports Science, Juntendo University, Chiba 270-1695, Japan; 9 Mito Medical Center, Tsukuba University Hospital, Ibaraki 310-0015, Japan

**Keywords:** locomotive disorders, daily step count, moderate to vigorous physical activity, triaxial accelerometer, aging

## Abstract

This study aimed to evaluate the association between locomotive syndrome (LS) and daily physical activity (PA) in community-dwelling older adults. This cross-sectional study included 80 healthy Japanese older adults (40 men and 40 women; age: 60–79 years). Habitual daily PA was evaluated using a triaxial wrist accelerometer. Participants were divided into two groups based on the results of the two-step test, stand-up test, and 25-question geriatric locomotive function scale. Binomial logistic regression analysis was conducted to examine the statistical relationships between daily PA and category of LS, adjusting for age from adjusted odds ratio (adjusted OR) with the 95 percent confidence intervals (95%CI) and bootstrap 95%CI. The mean step count and time spent on moderate to vigorous physical activity (MVPA) were significantly higher among non-LS participants than among LS participants in women, but not in men. Logistic regression analyses indicated that spending longer than 28 min/day on MVPA was significantly associated with a lower likelihood of LS relative to short time category under 28 min/day in women (adjusted OR = 0.12, 95%CI = 0.02–0.59, bootstrap 95%CI = 0.01–0.43), but not in men. This study suggests that in community-dwelling older women, those with higher MVPA had lower odds of LS, and daily MVPA was associated with LS, but not in men. Therefore, the associations between LS and daily physical activity were partly dependent on sex differences.

## 1. Introduction

Aging is associated with a decline in physical function, particularly in balancing, climbing stairs, and standing up from a sitting position [1,2,3,4]. Skeletal muscles, one of the locomotory organs, are known to decline with age [5]. Age-related muscle weakness (sarcopenia) is considered a cause of decreased activities of daily living (ADL), as well as an increased risk of falls and fractures, leading to reduced independence [6,7]. Japan has one of the highest life expectancies in the world, and the Japanese population is aging rapidly. The aging rate of Japan exceeded 28.8% in 2020 [8], which can be considered a “super-aged society”, since one out of four Japanese is older than 65 years [9]. As older adults suffer from many diseases and experience disability in ADL due to functional limitations, it is necessary for health care professionals to provide support, not only to control diseases but also to restore or maintain physical function and prevent or reduce disability [9].

Locomotive syndrome (LS) is a term proposed by the Japanese Orthopedic Association (JOA) to define a state of degraded mobility due to impaired locomotive organs [9]. This syndrome is caused by weakness and fragility of the function of the musculoskeletal organs, such as the bones, joints, and muscles, leading to difficulties in the ability to stand, walk, run, climb stairs, and perform other physical functions essential to daily living and mobility. Sarcopenia is also a locomotor disorders that can cause LS [9]. Methods for evaluating LS have been established by the JOA, including two functional tests and a questionnaire: the stand-up test, two-step test, and 25-question geriatric locomotive function scale for assessing the risk of LS [9]. Previous studies showed that the diagnosis of LS is made using these three tests, which are based on age-dependent changes in adults without specific mobility disorders aged 20–91 [7,10]. Moreover, Ogata et al. [10] found that this battery of tests is an effective screening tool in determining the risk of LS in adults. In the last decade, the awareness of LS has spread considerably throughout Japan and recently, it has also started to spread internationally [4,7,9,10,11,12,13]. The aging of society in any country will result in significant problems, similar to those currently experienced in Japan. Therefore, the initiation and advancement of countermeasures for LS in Japan are likely to set a precedent that will become meaningful for many countries in the future [9].

The prevalence and complication rates of LS, sarcopenia, and frailty were studied in 963 Japanese individuals (321 men and 642 women) aged 60 years or older [11,12]. According to the results, the prevalence of LS, sarcopenia, and frailty was 81.0%, 8.7%, and 4.5%, respectively. Furthermore, of the 84 patients with sarcopenia and 43 with frailty, 83 (98.8%) and 43 (100%) had locomotor complications, respectively, suggesting that the decline in mobility function represented by locomotion may be a precursor to sarcopenia and frailty. Imagama et al. [13] suggested that compared to frailty, LS had a stronger correlation with musculoskeletal factors in the development of poor physical and mental quality of life. These factors should be evaluated earlier, especially in independent older adults, to maintain their ADL and quality of life. Therefore, risk factors for LS include decreased levels of daily physical activity and ADL that occur with aging. To maintain ADL and the quality of life of the senior generation, specific target values for daily physical activity should be provided in combination with LS screening. However, the quantity and quality of daily physical activity associated with the components of LS have yet to be clearly defined.

The intensity of physical activity shows a positive association with various components of physical fitness and function, as well as negative associations with the incidence of diseases that cause morbidity and mortality in older adults [14,15,16]. In particular, an intensity above three metabolic equivalents is essential for successful aging [17]. Previous studies demonstrated that the daily step count and moderate to vigorous physical activity (MVPA) were associated with musculoskeletal tissue health, calcaneal health, and frailty status [3,6,16,18,19]. A recent study showed that walking speed was positively correlated with MVPA in older women with LS, but not in those without LS [20], suggesting that slow walking speed in older women with LS occurs in connection with lower MVPA. Park et al. [18] demonstrated that older adults aged 65–84 years who walked at least 7000–8000 steps/day and/or spent 15–20 min/day performing MVPA using a uniaxial accelerometer were likely to develop muscle mass above the sarcopenia threshold of clinical diagnosis.

Wearable sensors have the benefits of objectivity, portability, and affordability, making these devices useful as primary prevention tools for LS and sarcopenia in the home and community. Although the reference value for daily physical activity has been shown in sarcopenia to be on a clinical and pathological basis [18], the target value for LS, which is not on a pathological basis, has not yet been shown. Regarding the primary prevention and improvement of LS, it is important to indicate the reference value of daily physical activity parameters in community-dwelling older men and women with/without LS. The study by Nishimura et al. [20] included only older women, and the assessment of LS was based only on stand-up tests. Moreover, a history of resistance training and other sports activity (i.e., tennis, golf, cycling, etc.) was not included in the exclusion criteria of the study. Among community-dwelling older men and women with no history of resistance training and other sports activity, the association between the presence of LS and habitual daily physical activity characteristics is unclear. Therefore, this study aimed to evaluate the association between LS and daily physical activity in community-dwelling older adults. We hypothesized that the amount of daily physical activity would be lower in older men and women with LS than in those without LS, and LS would be associated with MVPA. The primary outcomes were the mean value of the daily step counts and activity time based on exercise intensities in older men and women with respect to LS.

## 2. Materials and Methods

### 2.1. Participants

The study participants included healthy, older, community-dwelling Japanese participants in Inzai, Sakura, Shisui, Narita and Tomisato city in Chiba. All participants were recruited through printed media, such as recruitment flyers or posters that were distributed or displayed in the community or public facilities. One hundred forty-three older community-dwelling Japanese participated in the briefing session. In addition, the participants completed a self-report questionnaire regarding medical history and comorbid conditions. We excluded individuals who were unable to follow our instructions and those with chronic orthopedic conditions or any health or medical condition that limited their ability to undertake light-to-moderate walking, as well as those with a history of resistance training, cycling, and other sports activities (i.e., tennis, golf, cycling, etc.) for at least one year prior to the start of the study. Of these participants, we excluded participants who had a history of resistance training, cycling, and other sports activity (*n* = 44), participated in other intervention studies (*n* = 11), as well as were aged more than 80 (*n* = 4) and less than 60 (*n* = 4). Ultimately, 80 healthy, Japanese community-dwelling older adults (40 men and 40 women; age: 60–79 years) were included in this cross-sectional study. Before the study began, participants provided written consent to participate after receiving information about the procedures and purpose of the study. This study was approved by the Ethics Committee of the Juntendo University (Approval Number: 26–75).

### 2.2. Measurements

#### 2.2.1. Tests and Definition of Locomotive Syndrome

To screen the presence of LS, the participants were tasked to perform the two-step test and stand-up test as functional tests [10], and were asked to answer the 25-question geriatric locomotive function scale (GLFS-25) questionnaire [21]. Before tests, participants underwent a blood pressure measurement to check whether they were in a hypertensive state or not. Subsequently, anthropometrics and body composition measurements were performed, followed by a warm-up by stretching. A 3-to-5-min break was taken between each test.

For the two-step test, participants stood with the toes of both feet behind a starting line. They were instructed to take two long strides (as long as possible) and to align both feet. The length of the two strides was measured from the starting line to the tips of the toes. The two-step test score was calculated using the following formula: length of the two strides (cm)/height (cm).

For the stand-up test, participants were instructed to stand up using one or both legs from a specific height. Seats were placed at the following heights: 40, 30, 20, and 10 cm. Participants stood up from each seat (in descending height order) using each leg separately. Participants passed the test at a specific height if they were able to stand up without leaning back to gain momentum and maintain the posture for 3 s. If participants were unable to stand up on one leg (right and left separately) from a height of 40 cm, they were challenged to stand up on both legs from a height of 10 cm. A score from “0” to “8” was allocated based on the difficulty, as described by Ogata et al. [10].

The GLFS-25 is a self-administered, comprehensive questionnaire consisting of 25 items that assess pain (4), ADL (16), social function (3), and the status of mental health (2) of patients during the previous month. These items are graded using a five-point scale from 0 (no impairment) to 4 points (severe impairment). The scores are added up to obtain a total score (minimum = 0, maximum = 100), with a higher score associated with worse locomotive function. Our previous study [22] indicated that the measured variables from the stand-up test, two-step test, and GLFS-25 have enough validity and reliability, with intra-class correlation coefficients of 0.87, 0.93, and 0.76, as well as Cronbach’s α of 0.93, 0.95, and 0.88, respectively. These tests were used to assess declines in mobility for each participant.

LS was evaluated using the LS risk tests, namely the two-step test, stand-up test, and GLFS-25. Participants were classified as having LS if they fulfilled one or more of the following criteria: (1) difficulty in standing up from a seat at a height of 40 cm using one leg in the stand-up test (either leg), (2) a two-step test score < 1.3, and (3) a GLFS-25 score ≥ 7 [9]. Participants were divided into LS and non-LS groups based on the criteria of the JOA definition.

#### 2.2.2. Habitual Daily Physical Activity

We measured daily physical activity using a small and lightweight (20 mm × 39 mm × 14 mm, 20 g) triaxial accelerometer (UW-301, A&D Co., Ltd., Tokyo, Japan). Participants were instructed to wear the accelerometer around the wrist continuously for 9–14 days continuously while performing their daily activities, except when sleeping or bathing. After 14 days, the number of daily steps determined by the accelerometer was obtained. The accelerometer was not reset throughout the study, and the participants noted down the displayed number at the beginning and end of each day. The obtained data were then downloaded to a computer. The accelerometer readings recorded for a minimum of seven consecutive days, excluding the distribution and collection days, were considered in the study. Days wherein the accelerometer was not worn for more than two hours were excluded [23]. Activities were classified into five levels of intensity, according to the accelerometer data: (1), resting (<1.1 metabolic equivalents (METs)); (2), sitting behavior (1.1–1.4 METs); (3), standing behavior (1.5–2.9 METs); (4), moderate physical activity (3.0–5.9 METs); and (5), vigorous physical activity (≥6 METs). The sum of the time spent in moderate and vigorous physical activity (MVPA: ≥3 METs) was calculated and defined. Additionally, the sum of the time spent in resting and sitting behaviors (RSB: <1.5 METs) was calculated.

#### 2.2.3. Anthropometrics and Body Composition

Anthropometric measurements included height (cm), weight (kg), and body mass index (BMI, weight [kg]/height [m^2^]). Body composition measurements included body fat, muscle mass, and waist-to-hip ratio, which were estimated by bioelectrical impedance analysis using a body composition analyzer (InBody 730, Biospace Co., Ltd., Seoul, Korea). Participants were instructed to finish their last meal at least four hours before the experimental session and to empty their bladder before these measurements. They were evaluated in their underwear and were asked to stand barefoot on toe and heel electrodes and to hold the handgrips with their arms hanging down a few centimeters from the hip [24]. Appendicular skeletal lean mass (ALM) was calculated as the sum of the muscle mass of the arms and legs [25]. We calculated the skeletal muscle index (SMI) as follows: SMI = ALM/height^2^. Leg-SMI was calculated as the sum of the muscle mass of both legs.

#### 2.2.4. Maximal Isometric Strengths of Leg Muscle

The maximal voluntary isometric strength of the knee extensors was determined using a dynamometer (Takei, Tokyo, Japan). Each participant was seated on a chair with the hip joint angle at 90° flexion (0° = full hip extension). Prior to the test, several warm-up contractions (2–3 submaximal contractions and 1–2 near-maximal contractions) were performed. They were instructed to perform maximum isometric knee extensions two or three times. The best-recorded value was used as the representative, and the weight-bearing index (knee strength/body weight; KE-WBI) was calculated. This measurement was conducted at the end of the physical function tests. During the measurement, a break of about 30 to 60 s was taken between each time. However, five participants were unable to perform the test due to high blood pressure that was measured before conducting the test. With regard to knee extension strength, the test–retest (inter-session) reliabilities using intraclass correlation coefficients, standard error of the mean, and minimal difference were 0.945, 3.41 kg, and 9.45 kg, respectively.

### 2.3. Statistical Analysis

Data are presented as means ± standard deviations (SD). Unpaired *t*-tests were used to test the significance of differences in measured variables between men and women, and between non-LS and LS in each sex group. When a significant F-value was observed (age and GLFS-25 in men, and age, height and GLFS-25 in women), Welch’s *t*-test were conducted to determine group differences in non-LS and LS. Effect sizes (Cohen’s *d*) were also calculated, with values of 0.2, 0.5, and 0.8 representing a small, moderate, and large difference, respectively [26]. The partial correlation coefficient adjusted for age was used to determine the association among the three LS parameters, daily physical activity, and KE-WBI by men and women. The partial correlation coefficient adjusted for age was used to determine the association between the three LS parameters, daily physical activity, and KE-WBI in men and women. Participants were divided into two groups based on step counts, RSB, and MVPA values by sex (low vs. high). Subsequently, binomial logistic regression analysis was conducted to examine the statistical relationships between daily PA and category of LS adjusting for age from adjusted odds ratio (adjusted OR) with the 95 percent confidence intervals (95%CI). Additionally, bootstrap resampling (times = 1000) was used to enhance internal validity and calculate 95%CIs from the binomial logistic regression analysis. All analyses were performed using SPSS software (ver. 27; IBM Corp., Armonk, NY, USA). Statistical significance was set at *p* < 0.05.

## 3. Results

Overall, the prevalence of LS was 51.3% (41/80; men, 20; women, 21). The non-LS group included 20 men and 19 women. The characteristics of participants in the non-LS and LS groups are presented in Table 1. Among the participants, the proportions of those who obtained a two-step test score < 1.3, had difficulty in standing up from a seat at a height of 40 cm using one leg in the stand-up test, and a GLFS-25 score ≥ 7 were 12.5%, 36.3%, and 21.3%, respectively. The percentage of body fat in the LS women was significantly higher than that in non-LS participants (*t* (38) = −2.28, *p* = 0.03, *d* = 0.72, moderate, *F* = 1.54); however, no difference was observed in men (*t* (38) = −1.84, *p* = 0.07, *d* = 0.58, moderate, *F* = 0.39). Men in the LS group were significantly taller and heavier than those in the non-LS group (height, *t* (38) = −2.56, *p* = 0.01, *d* = 0.81, large, *F* = 0.23; weight, *t* (38) = −2.56, *p* = 0.01, *d* = 0.81, large, *F* = 0.51). The KE-WBI of men and women in the LS group was significantly lower than that of those in the non-LS group (men, *t* (37) = 3.25, *p* = 0.00, *d* = 1.04, large, *F* = 0.29; women, *t* (34) = 2.52, *p* = 0.02, *d* = 0.84, large, *F* = 0.88).

The daily physical activity parameters of participants in the non-LS and LS groups are presented in Table 2. The time spent in RSB was significantly shorter in women than in men (*t* (78) = 3.17, *p* = 0.00, *d* = 0.70, moderate, *F* = 0.02). However, no significant differences were observed between the sexes in the mean step count (*t* (78) = 1.47, *p* = 0.14, *d* = 0.33, small, *F* = 0.47) and time spent on MVPA (*t* (78) = 1.94, *p* = 0.06, *d* = 0.44, small, *F* = 2.66). For women, the mean step count and time spent on MVPA were significantly lower in the LS group than in the non-LS group (*t* (38) = 2.58, *p* = 0.01, *d* = 0.82, large, *F* = 0.19; *t* (38) = 3.77, *p* = 0.00, *d* = 1.19, large, *F* = 2.63). On the other hand, the time spent on RSB was longer in women in the LS group than in those in the non-LS group (*t* (38) = −2.26, *p* = 0.03, *d* = 0.72, moderate, *F* = 0.10). However, there were no significant differences between the non-LS and LS groups with respect to physical activity parameters in men.

Table 3 shows the partial correlation coefficient adjusted for age between daily physical activity (step/day, RSB, and MVPA) and there LS tests score or KE-WBI of older men and women. For women, the RSB was significantly correlated with the stand-up test score (r = −0.35; *p* = 0.04). Additionally, the MVPA in women was significantly correlated with the KE-WBI (r = 0.39; *p* = 0.02); however, no significant correlations were observed in men.

Table 4 shows the results of the binomial logistic regression analyses. Crude OR and adjusted OR with 95%CI and adjusted OR with bootstrap 95%CI represent the strength of the relationships between daily physical activity and category of LS. In the present analysis, the effects of sex difference and age were adjusted. As a result, spending time longer than 28 min/day on MVPA was significantly associated with a lower likelihood of LS relative to the short time category under 28 min/day (adjusted OR = 0.12, 95%CI = 0.02–0.59, bootstrap 95%CI = 0.01–0.43). However, no significant association was observed in men.

## 4. Discussion

This study aimed to evaluate the association between LS and daily physical activity in community-dwelling older adults. In our study, the prevalence of LS was 51.3% (41/80; men, 20; women, 21). The main findings of this study are as follows: (1) Older women with LS had lower daily physical activity (step count and time spent on MVPA) and relative knee extension strength (KE-WBI) than those without. (2) Binomial logistic regression analyses indicated that LS was associated with MVPA (≥3 METs) in older women.

Regular physical activity can delay the age-related deterioration of functional capacity, independent of the presence of other health problems [1,27,28]. With regard to the association between physical activity and LS, Nishimura et al. [29] suggested that the frequency of physical activity of participants aged between the ages of 25 and 50 years was significantly related to LS, among which, those who reported no regular exercise habits between the ages of 25 and 50 years had the highest prevalence of LS. Furthermore, we found that the daily physical activity of women participants with LS, particularly the step count and time spent on MVPA, was significantly lower than that of women participants without LS. Binomial logistic regression analyses indicated that LS was associated with MVPA (≥3 METs) in older women, not men. Aoyagi et al. [30] showed that lower-extremity functions such as gait speed and knee extension torque were more closely associated with the daily step count and MVPA in older women, suggesting that indices of lower extremity function are significantly greater in older individuals who are physically more active. Thus, it is suggested that older women with lower levels of daily physical activity may be at increased risk for LS due to the weakening and loss of musculoskeletal tissues [9,11,12]. In the present study, binomial logistic regression analyses were conducted to examine the relationship between daily PA and the category of LS, adjusting for age in men and women. We found that spending time on MVPA longer than 28 min/day was associated with a lower likelihood of LS compared to performing MVPA under 28 min/day in women. Park et al. [18] reported that older adults aged 65–84 years who spent 15–20 min/day performing MVPA were likely to develop muscle mass above the sarcopenia threshold of clinically diagnosis. As LS is not caused on a pathological basis, comparisons of judgment criteria indicate that LS will probably be detected earlier than frailty and sarcopenia [9,11,12]. Although the causal relationship between daily physical activity and LS risk is unclear, our results suggest an association between daily MVPA and LS.

Our results showed that there were no significant differences observed between the non-LS and LS groups with respect to daily physical activity parameters such as step count, RSB, and MVPA in men. Moreover, there was no association between LS and daily physical activity. Although the mechanism underlying the association between physical activity and the prevalence of LS in men in our cross-sectional study is unclear, the possible factors have been discussed previously. One potential factor could be the body status; men in the LS group were significantly taller and heavier than those in the non-LS group. With regard to the stand-up test, Miyamoto et al. [31] reported that the stand-up test was significantly affected by height, and taller participants showed a higher ratio of LS to Level 1 according to JOA guidelines and LS was defined as participants being unable to stand up on one leg from a seat of 40 cm high. When using the same chair with a height of 40, 30, 20, and 10 cm, regardless of the participant’s height, taller participants needed to make bigger movements when performing the transition from sitting to standing. A previous study indicated that a lower seat brings down the center of gravity, as well as increases the degree of trunk flexion and angular displacement of the trunk, hip, knee, and ankle [32]. Therefore, when an individual is taller or heavier, the load on the body increases, and the incidence of LS might be higher regardless of daily physical activity. Therefore, future study is needed to examine the association between daily physical activity and muscle function adjusted by height and weight.

In addition, there was no significant difference in muscle mass, SMI, and leg-SMI in men (Table 1). Recent studies have shown that appendicular muscle mass and SMI of older women were almost the same between LS and non-LS groups [20,33]. Therefore, it could be possible that LS was not associated with muscle mass and SMI using bioelectrical impedance analysis in older individuals aged around 70 years. Recently, Natsume et al. [34] reported that the muscle thickness of the anterior thigh and rectus abdominis was significantly lower in older men with LS, and the reduction in site-specific muscle mass significantly correlated to LS-related physical functions. Previous studies on LS also demonstrated that 10 m gait speed and muscle strength of the legs were significantly lower in the older adults with LS than in those without [33,35]. Similarly, our results showed that the KE-WBI of women with LS was significantly lower than that in the non-LS group. Given the above findings, LS is associated with site-specific muscle mass, such as the anterior thigh and rectus abdominis, or musculoskeletal function and physical performance, rather than quantitative aspects such as whole body or limb muscle mass.

It is well known that absolute muscle mass [18,25] and absolute muscle strength [36,37] are generally lower in women than in men. Omori et al. [38] reported that a significant decline in the quadriceps muscle strength was observed in individuals in their sixties and seventies. Additionally, the level of muscle strength of women in their fifties, sixties, and seventies was significantly lower than that of men. To support these points, we found that the KE-WBI of older individuals aged 60–79 years was lesser in women than in men. Therefore, a decrease in activity and mobility may be observed earlier in women than in men. In the present study, the percentage of body fat was higher in the LS group than in the non-LS group, and the RSB was negatively correlated with the stand-up test score (r = −0.35; *p* = 0.04) in women. A low level of daily physical activity reduces mechanical stimulus to the musculoskeletal system, thus increasing the risk of LS [29]. A previous study reported that RSB can cause muscle weakness and impaired gait function, resulting in an increased risk of LS and frailty [13]. Moreover, long periods of inactivity may cause fat accumulation, muscle atrophy, and increased central obesity [39]. Since older Japanese women traditionally spend long periods of time performing low-intensity household tasks [40], a decrease in the resting and sitting time may be important. Although the present study cannot ascertain the causality due to the cross-sectional data, we found that maintaining the quadriceps muscle strength was essential in daily life. In this regard, we recommend that older women with LS practice an interval or progressive walking program, which has been shown to improve leg muscle strength [41]. The research evidence and exercise protocols will be beneficial to increasing intentional MVPA and preventing LS in older women.

To the best of our knowledge, our study is the first to examine the association between LS and daily physical activity in community-dwelling older adults using a triaxial accelerometer. However, the present study has several limitations. First, this was a cross-sectional study; therefore, further cohort studies are warranted to establish a relationship between daily physical activity and LS. Second, the participants were not randomly selected from those cities in our study; they may have been more health conscious than the general population, suggesting that selection bias might have been present. Therefore, further research should be conducted with randomized sampling. Third, the sample size of 80 was small compared to previous studies of LS, sarcopenia, and frailty with big sample sizes [10,11,12,13,18,31,39]. We used bootstrapping methods in logistic regression analyses to provide reliable data in a small sample size; however, further studies with a larger sample size are required to confirm our data. Fourth, our participants were limited to relatively healthy older individuals aged 60–79 years who had no history of resistance training, cycling, and other sports activities (i.e., tennis and cycling, etc.); hence, the results of this study are limited to the older population who have no habits of exercise. Therefore, a future follow-up study is required to clarify the causal relationships among these factors.

## 5. Conclusions

This study suggests that in community-dwelling older women, those with higher MVPA had lower odds of locomotive syndrome, and daily MVPA was associated with locomotive syndrome, but not in men. Therefore, the associations between locomotive syndrome and daily physical activity were partly dependent on sex differences. These findings may contribute to the treatment and primary prevention of older women at risk of locomotive syndrome.

## Figures and Tables

**Table 1:** Characteristics of the study participants.

Variables		Total	Non-LS	LS	ES (*d*) ^a^
Age (years)	Men	69.9 ± 4.8	68.6 ± 3.4	71.2 ± 5.6	0.56
	Women	68.7 ± 4.5	66.9 ± 4.0	70.3 ± 4.3 ^††^	0.82
Height (cm)	Men	165.8 ± 6.4	163.4 ± 5.7	168.2 ± 6.3 ^††^	0.81
	Women	152.9 ± 5.5 **	152.8 ± 3.5	153.0 ± 7.0	0.04
Weight (kg)	Men	65.2 ± 9.3	61.7 ± 8.5	68.7 ± 8.9 ^††^	0.81
	Women	52.7 ± 7.3 **	51.0 ± 7.1	54.1 ± 7.3	0.43
BMI (kg/m^2^)	Men	23.7 ± 2.5	23.1 ± 2.6	24.2 ± 2.4	0.46
	Women	22.5 ± 2.8	21.9 ± 2.9	23.1 ± 2.7	0.43
Body fat (%)	Men	23.5 ± 5.4	22.0 ± 4.8	25.0 ± 5.5	0.58
	Women	31.7 ± 5.9 **	29.5 ± 6.3	33.6 ± 5.0 ^†^	0.72
Muscle mass (kg)	Men	27.3 ± 3.2	26.4 ± 2.9	28.2 ± 3.4	0.57
	Women	18.9 ± 2.1 **	19.0 ± 1.9	18.8 ± 2.3	0.07
SMI (kg/m^2^)	Men	7.56 ± 0.61	7.43 ± 0.67	7.70 ± 0.52	0.46
	Women	6.02 ± 0.59 **	6.01 ± 0.57	6.02 ± 0.62	0.02
Leg-SMI (kg/m^2^)	Men	5.64 ± 0.73	5.53 ± 0.52	5.76 ± 0.41	0.48
	Women	4.65 ± 0.43 **	4.62 ± 0.38	4.68 ± 0.47	0.14
KE-WBI (kg/kg weight)	Men	0.74 ± 0.17	0.82 ± 0.18	0.66 ± 0.13 ^††^	1.04
	Women	0.64 ± 0.13	0.70 ± 0.13	0.60 ± 0.11 ^†^	0.84
Stand-up test (score)	Men	4.53 ± 1.18	5.30 ± 0.73	3.75 ± 1.02 ^††^	1.75
	Women	4.65 ± 1.08	5.37 ± 0.60	4.00 ± 1.00 ^††^	1.64
Two-step test (score)	Men	1.44 ± 0.11	1.48 ± 0.11	1.40 ± 0.10 ^††^	0.82
	Women	1.36 ± 0.12	1.43 ± 0.09	1.31 ± 0.12 ^††^	1.09
GLFS-25 (score)	Men	4.28 ± 3.73	2.37 ± 1.83	6.00 ± 4.18 ^††^	0.73
	Women	3.90 ± 4.31	2.40 ± 1.98	5.40 ± 5.43 ^†^	1.11

Means ± standard deviations; ** *p* < 0.01, vs. Men; ^†^ *p* < 0.05, ^††^ *p* < 0.01, vs. non-LS in each sex group. ^a^: non-LS vs. LS; LS: locomotive syndrome group, non-LS: non-locomotive syndrome group, ES: Effect Size., BMI: body mass index; SMI: skeletal muscle index., Leg-SMI: leg muscle mass index; KE-WBI: knee extension strength/body weight; GLFS-25: 25-question geriatric locomotive function scale.

**Table 2:** Daily physical activity of the participants in this study.

Daily PA Variables		Total	Non-LS	LS	ES (*d*) ^a^
Step count (steps/day)	Men	6807 ± 3410	7258 ± 3881	6356 ± 2895	0.26
	Women	5819 ± 2519	6829 ± 2390	4905 ± 2321 ^††^	0.82
Time spent on RSB (min/day)	Men	1094.8 ± 78.6	1082.2 ± 67.9	1107.4 ± 88.0	0.32
	Women	1038.3 ± 83.6 **	1008.4 ± 78.1	1065.3 ± 80.7 ^†^	0.72
Time spent on MVPA (min/day)	Men	40.7 ± 25.4	42.1 ± 28.1	39.3 ± 23.1	0.11
	Women	31.2 ± 17.6	40.7 ± 18.0	22.6 ± 12.2 ^††^	1.19

Means ± standard deviations, ** *p* < 0.01, vs. Men, ^†^ *p* < 0.05, ^††^ *p* < 0.01, vs. non-LS in each sex group. ^a^: non-LS vs. LS., LS: locomotive syndrome group; non-LS: non-locomotive syndrome group; ES: Effect Size; PA: physical activity; RSB: resting and sitting behaviors (<1.5 METs); MVPA: moderate to vigorous physical activity (≥3 METs); METs: metabolic equivalents.

**Table 3:** Partial correlation coefficient adjusting for age between daily physical activity and locomotive syndrome tests score or maximal isometric strengths of leg muscle in older men and women.

Daily PA Variables	Stand-Up Test	Two-Step Test	GLFS-25	KE-WBI
Men (*n* = 40)				
Step count	−0.08	0.16	−0.22	−0.01
Time spent on RSB	−0.06	−0.27	0.27	−0.04
Time spent on MVPA	−0.05	0.19	−0.28	−0.05
Women (*n* = 40)				
Step count	0.23	0.05	−0.15	0.18
Time spent on RSB	−0.35 *	−0.14	0.04	−0.31
Time spent on MVPA	0.26	0.11	−0.25	0.39 *

* *p* < 0.05, PA: physical activity, KE-WBI: knee extension strength/body weight, RSB: resting and sitting behaviors (<1.5 METs), MVPA: moderate to vigorous physical activity (≥3 METs), METs: metabolic equivalents.

**Table 4:** Binomial logistic regression analyses of daily physical activity of older men and women in the locomotive syndrome and non-locomotive syndrome groups.

	Crude OR	*p*-Value	Adjusted OR	*p*-Value	Adjusted OR	*p*-Value
	(95%CI)		(95%CI)		(Bootstrap CI)	
Men						
Step count (step/day)						
Low (≤6112.5)	Reference		Reference		Reference	
High (>6112.5)	0.29 (0.08–1.06)	0.062 ^†^	0.34 (0.09–1.31)	0.117	0.34 (0.05–1.35)	0.133
RSB (min/day)						
Low (≤1096.9)	Reference		Reference		Reference	
High (>1096.9)	1.49 (0.43–5.19)	0.528	1.72 (0.46–6.45)	0.419	1.72 (0.45–9.55)	0.430
MVPA (min/day)						
Low (≤39.85)	Reference		Reference		Reference	
High (≥39.86)	0.44 (0.13–1.58)	0.209	0.45 (0.12–1.68)	0.236	0.45 (0.08–1.78)	0.256
Women						
Step count (step/day)						
Low (≤5511.5)	Reference		Reference		Reference	
High (>5511.5)	0.23 (0.06–0.87)	0.030 *	0.28 (0.07–1.15)	0.076 ^†^	0.28 (0.03–1.23)	0.080 ^†^
RSB (min/day)						
Low (≤1032.35)	Reference		Reference		Reference	
High (>1032.35)	0.55 (0.16–1.91)	0.344	1.64 (0.42–6.35)	0.477	1.64 (0.38–9.70)	0.468
MVPA (min/day)						
Low (≤27.99)	Reference		Reference		Reference	
High (≥28.01)	0.14 (0.04–0.58)	0.006 **	0.12 (0.02–0.59)	0.009 **	0.12 (0.01–0.43)	0.003 **

^†^ *p* < 0.10; * *p* < 0.05; ** *p* < 0.01; Adjusted OR: Odds ratio adjusted by age; 95%CI: 95% confidence intervals; Bootstrap CI: 95% confidence intervals estimated by 1000 bootstrap samples; RSB: time spent on resting and sitting behaviors (<1.5 METs); MVPA: time spent on moderate to vigorous physical activity (≥3 METs); METs: metabolic equivalents.

## Data Availability

Data presented in this study are available on request from the corresponding author. Data are not publicly available due to privacy.
