# Correction: Ishihara et al. Association between Daily Physical Activity and Locomotive Syndrome in Community-Dwelling Japanese Older Adults: A Cross-Sectional Study. *Int. J. Environ. Res. Public Health* 2022, *19,* 8164

**DOI:** 10.3390/ijerph20186751

**Published:** 2023-09-13

**Authors:** Yoshihiko Ishihara, Hayao Ozaki, Takashi Nakagata, Toshinori Yoshihara, Toshiharu Natsume, Tomoharu Kitada, Masayoshi Ishibashi, Pengyu Deng, Yasuyuki Yamada, Hiroyuki Kobayashi, Shuichi Machida, Hisashi Naito

**Affiliations:** 1 School of Science and Technology for Future Life, Tokyo Denki University, Tokyo 120-8551, Japan; 2 Faculty of Health Sports Science, Juntendo University, Chiba 270-1695, Japan; 3 School of Sport and Health Science, Tokai Gakuen University, Miyoshi 470-0207, Japan; 4 Department of Physical Activity Research, National Institutes of Biomedical Innovation, Health and Nutrition (NIBIOHN), Tokyo 162-8636, Japan; 5 School of Medicine, Tokai University, Isehara 259-1193, Japan; 6 COI Project Center, Juntendo University, Tokyo 113-8421, Japan; 7 Faculty of Business Administration, Seijoh University, Miyoshi 476-8588, Japan; 8 Graduate School of Health and Sports Science, Juntendo University, Chiba 270-1695, Japan; 9 Mito Medical Center, Tsukuba University Hospital, Ibaraki 310-0015, Japan

## Text Correction

There was an error in the “*d*-value of the first paragraph in Section 3 (Results)” in the original publication (Page 5, Line 41) [1]. We mentioned that “The percentage of body fat in the LS women was significantly higher than that in non-LS participants (*t* (38) = −2.28, *p* = 0.03, *d* = 1.54, large).” Also, the *F*-value was not described.

A correction was made to “The percentage of body fat in the LS women was significantly higher than that in non-LS participants (*t* (38) = −2.28, *p* = 0.03, *d* = 0.72, moderate, *F* = 1.54);”.

There was an error in the “sentence in the first paragraph in Section 3 (Results)” in the original publication (Page 5, Lines 42–47) [1]. The *F*-value was not described.

A correction was made to “however, no difference was observed in men (*t* (38) = −1.84, *p* = 0.07, *d* = 0.58, moderate, *F* = 0.39). Men in the LS group were significantly taller and heavier than those in the non-LS group (height, *t* (38) = −2.56, *p* = 0.01, *d* = 0.81, large, *F* = 0.23; weight, *t* (38) = −2.56, *p* = 0.01, *d* = 0.81, large, *F* = 0.51). The KE-WBI of men and women in the LS group was significantly lower than that of those in the non-LS group (men, *t* (37) = 3.25, *p* = 0.00, *d* = 1.04, large, *F* = 0.29; women, *t* (34) = 2.52, *p* = 0.02, *d* = 0.84, large, *F* = 0.88).”

There was an error in a “sentence of the second paragraph in Section 3 (Results)” in the original publication (Page 6, Line 9) [1]. We mentioned that “For women, the mean step count and time spent on MVPA were significantly lower in the LS group than in the non-LS group (*t* (38) = 3.77, *p* = 0.00, *d* = 2.63, large, *F* = 2.63).”

A correction was made to “For women, the mean step count and time spent on MVPA were significantly lower in the LS group than in the non-LS group (*t* (38) = 2.58, *p* = 0.01, *d* = 0.82, large, *F* = 0.19; *t* (38) = 3.77, *p* = 0.00, *d* = 1.19, large, *F* = 2.63).”

The authors apologize for any inconvenience caused and state that the scientific conclusions are unaffected. This correction was approved by the Academic Editor. The original publication was also updated.
